# Similarity Simulation on the Movement Characteristics of Surrounding Rock and Floor Stress Distribution for Large-Dip Coal Seam

**DOI:** 10.3390/s22072761

**Published:** 2022-04-03

**Authors:** Wenxiang Cao, Honglin Liu, Yinjian Hang, Hongzhi Wang, Guodong Li

**Affiliations:** 1 College of Geology and Mines Engineering, Xinjiang University, Urumqi 830046, China; wenxiangcao@stu.xju.edu.cn (W.C.); wanghongzhi@xju.edu.cn (H.W.); cklgd2011@xju.edu.cn (G.L.); 2 Key Laboratory of Environmental Protection Mining for Minerals Resources at Universities of Education, Department of Xinjiang Uygur Autonomous Region, Xinjiang University, Urumqi 830047, China; 3 Xuzhou Mining Group Co., Ltd., Xuzhou 221000, China; hangyinjian@126.com

**Keywords:** pressure sensor, large dip, overburden migration, similarity simulation, asymmetric, close multi coal seams

## Abstract

The question of how to mine safely in close multi coal seams is the main concern for coal operators, in particular for large-dip coal seams with complex geological and mechanical conditions. This paper presents a detailed similarity simulation on the movement characteristics of the overburden and the stress distribution of underlying strata in terms of a specific coal mine in the Tielieke mining area of the Kubai coalfield via a three-dimensional photogrammetry system and a high-speed static resistance analyzer. The results show that the overburden strata are asymmetrically deformed around the coal pillar and the fracture area is perpendicular to the longwall with an “**M**” shape when deeper coal is mined. Moreover, the asymmetric movement of overburden results in the non-uniform distribution of stress on the floor of the coal pillar and ribs. In particular, stress is closely related to the location of the longwall, and stress of the coal pillar is much larger when it is closer to the deep side. The floor stress relief degree of the longwall in the deep zone is higher than that of its counterparts, providing a theoretical foundation for a reasonable layout and a support technique for roadways. The main contribution of this research that it can be used as a reference in maintaining the integrity of surrounding rock for large-dip coal seams with close distances.

## 1. Introduction

It has been well noted that coal provides an energy guarantee for China’s social development and economical construction [1]. With the gradual depletion of shallow coal resources in the eastern region, widely deposited large-dip coal seams in western China have gradually gained attention. The question of how to safely and efficiently excavate large-dip coal seams is becoming a key concern for coal operators [2]. A large-dip coal seam is defined as a coal seam with a dip angle within 35°~55°, which accounts for about 20% of verified deposits and 10% of total coal production in China. Attributed to the coal-forming environment and the geological conditions, the ore pressure law of large-dip coal seams is much more complex compared to its counterparts, and correspondingly, this type of seam is regarded as a difficult coal seam with respect to current mining techniques [3]. The tangential component of both the roof and floor increases along with the plane, while the vertical component decreases. As a result, the stress environment of a coal seam with a large dip is more complex than that of a coal seam with a small dip. Although the mining technology applied in near-horizontal and gently inclined coal seams is relatively mature, there is still a long way to go in achieving the safe and efficient excavation of large-dip coal seams. With the rapid development of intelligent sensors, this technology has attracted more and more attention of many scientific researchers. It is undoubtedly extremely beneficial to the improvement of the mining process of large-dip-angle coal seams. For example, stress sensors can monitor stress changes, and displacement sensors can monitor surrounding rock migration characteristics.

A great deal of theoretical research and field measurements on the fracture characteristics of overburden have been carried out for large-dip coal seams. Shaoxuan Hu et al. [4] systematically considered the influence of dip angle on the stability of surrounding rock and established the mechanical model termed “supporting–surrounding rock” in view of the problems with large dip angles, unstable equipment, low top coal recovery rates and difficulty in controlling surrounding rock. Honglin Liu et al. [5] proposed the concept of the damage coefficient based on a numerical simulation, based on which, asymmetric support technology was proposed and used in a practical application. Based on the thin plate theory, Ke Yang et al. [6] established the basic mechanical model of a roof and deduced a formula to calculate the basic stress distribution of a roof. They also investigated the basic deformation and failure criteria of a fully mechanized roof.

In addition, Dezhong Kong et al. [7,8,9,10,11,12,13,14] studied the distribution law of floor stress, roof fracture distribution, and large-dip coal seam fracture modes via a similarity simulation and numerical modeling. With the consideration of the asymmetric failure mechanism of overburden, Ding Lang et al. [15,16,17,18] proposed the stability control technology of roadway-surrounding rock. Yongping Wu, Panshi Xie et al. studied the time sequence characteristics of spatial migration and the collapse of surrounding rock in stopes and the mechanism of unbalanced ore pressure [19,20,21]. Honglin Liu et al. [22] analyzed the fracture and collapse characteristics of overburdened rock in a working face using two excavation methods of inverted mining and overhead mining. Liqiang Ma et al. [23] discussed the mechanical characteristics of strike support stability in the free state, support state, and a particular state of strike support along the working face with a large inclination dip. In addition, some scholars have studied the asymmetric movement law of overburden based on similar simulation experiments [24,25]. Jian Luo et al. [26] analyzed the distribution of concentrated stress in coal pillars and studied the stress distribution rule of the floor under coal pillars in close coal seams. Using a similarity simulation, Qing YE et al. [27] concluded that the stress increase zone was unbalanced and the characteristics of overburden movement—fissure—evolution in deep mines with large dips is very different. The problem of roadway support in large-dip coal seams has affected safe production in coal mines, and the development of new support structure has attracted more and more attention [28,29].

Scholars in other countries have carried out a lot of research on the mining of high-dip coal seams. Das, Arka Jyoti et al. [30,31] evaluated the strength of coal pillars in high-dip-angle coal seams through mathematical models and numerical simulations. The shear characteristics of high-dip-angle formations were mainly considered, and the research conclusions have important reference value for coal pillar strength in the process of high-dip-angle coal seam mining. Kulakov [32,33] conducted a systematic study on the geological mining conditions of high-dip coal seams. Partha Sarathi Paul et al. [34] studied the failure mechanism of inclined coal pillars, which is of great significance to the safe and efficient mining of coal resources. Garza-Cruz, T. et al. [35] established a calibration model that can effectively evaluate the effect of shear stress on coal pillar strength.

All of this above research mainly focuses on overburden fracture characteristics, theoretical calculation, and the stress distribution of single coal seams with large dips, rather than coal seams with close distance. To obtain in-depth understanding on the relationship between overburden movement characteristics and floor stress distribution of multi coal seams with large dips as well as the reasonable roadway layout of lower coal seams, a similarity simulation was carried out in this research. The simulation model was set up upon the typical geological conditions of Zhongwei coal mine in the Tieleke mining area of the Kubai coalfield. The stress distribution characteristics of the floor and the law of roof movement of large-dip coal seams were studied in detail. It is believed that the results obtained from this research will be basis for the rational layout of roadways under residual coal pillars in large-dip coal seams.

## 2. Mining Geological Condition

Zhongwei coal mine is located in the Kubai coal field in the Tieleke mining area, governed by Baicheng County, Aksu Region, Xinjiang. The overall structural form of the mining area is monoclinic with few faults, and there is only a small anticline structure at the western end. The strata trend east-north, with a dip of 135–165°, a dip of 25–55°, and an average of 35°. The anticline is vertically “S” shaped, extending in the NW~NW direction, and the anticline axis is inclined to the southeast, with an extension length of 2.2 km. As the burial depth increases, the anticline gradually transitions into a broad and gentle anticline. In the VI_13_ coal seam, the anticline extends in an NW direction with a length of about 1.5 km. The anticline axis is inclined to the southeast, with an inclination angle of about 35°.

**Figure 1:**
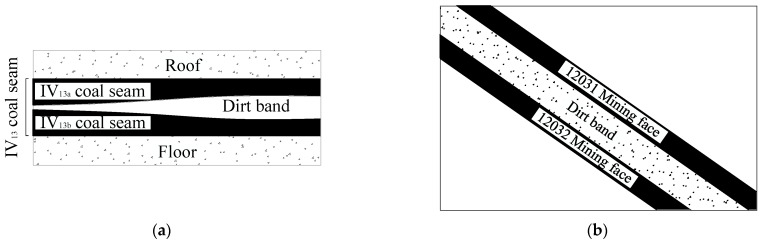
Spatial structure of coal seam. (**a**) Strike profile diagram; (**b**) inclination profile diagram.

The dip angle of the No. VI_13_ coal seam is around 25°~50° with the average thickness of 3.67 m. A comprehensive mechanized mining technique was adopted for the longwall, and some working faces adopt a pseudo-inclined arrangement. The average thickness of the VI_13_ coal seam in the second mining area is 5.5 m, with the gangue non-uniformly distributed in between. As depicted in Figure 1a, the coal seam consists of the upper and the lower layer (i.e., VI_13a_ and VI_13b_).

The buried depth of the 1203 working face is about 460~585 m, with the large dip angle ranging from 32° to 40°. The inclined length and the advanced distance of the working face are 162 m and 1245 m, respectively. The maximum thickness of the interlayer between coal seams is 4.2 m, while the thicknesses of coal seams in VI_13a_ and VI_13b_ are 1.7~2.7 m and 2.5~3.2 m, respectively. To facilitate the mining process, the upper and lower two stratified mining faces of 12031 and 12032 are arranged, which can be seen from Figure 1b. The main roof and floor slate petrological characteristics and the main physical and mechanical parameters of VI_13_ coal seam are listed in Table 1 and Table 2 for reference.

## 3. Experimental Program

### 3.1. Experimental Equipment

**Figure 2:**
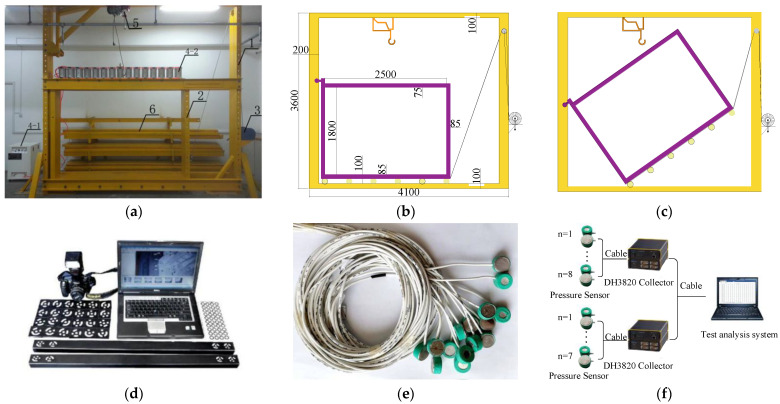
Servo loading experimental system for strata control in steep/thick coal seam. (**a**) System characteristics; (**b**) model frame parameters; (**c**) rotation model; (**d**) three-dimensional photogrammetry system; (**e**) pressure sensor; (**f**) stress monitoring equipment and process.

The “Steeply inclined/Thick Coal Seam Strata Control servo loading Experimental system” operated by Xinjiang University was used to carry out this research. The system is mainly composed of an outer frame (1), inner frame (2), spiral frame winch (3), and servo loading system (4), crane (5), fixing Plate (6) of the model frame, as shown in Figure 2a,b. The outer frame of the system is a model loading counter-frame, which is used to support and stabilize the inner frame of the model frame and fix the laying template. The inner frame is used for model laying, which can be rotated along with the left vertical plate and base of the outer frame of the model frame under the action of the frame winch. As shown in Figure 2c, the simulation of strata with different dip angles can be realized. The servo loading system is generally installed on the top of the inner frame, and is composed of a silent air pump, a pressure-regulating valve pipeline, and a cylinder group. In addition, a three-dimensional photogrammetry system was used to measure overburden displacement, as shown in Figure 2d. Moreover, a pressure sensor was used to monitor the floor stress distribution, which can be found in Figure 2e. The pressure sensor converts the stress change into an electronic signal, which is received by a DH3820 collector. The DH3820 collector transmits the stress changes of 15 pressure sensors to the test analysis system, as shown in Figure 2f.

### 3.2. Mix Design of Similarity Material

In accordance with the similarity criterion, the geometric similarity, motion similarity, boundary condition similarity, and corresponding physical quantity proportion had to remain constant [36,37]. Based on the mining geological conditions of Zhongwei coal mine and the similar material simulation experiment platform, the geometric similarity parameter C_L_ was determined as 1:100, while the time similarity C_t_ was set up as 1:10. Considering the measured rock density of 2.5 g·cm^−3^, the density of similar material was of 1.5 g·cm^−3^ with the similarity constant C_γ_ of 1:1.67. Herein, the stress and strength similarity were consistently C_σ_ = C_L_ × C_γ_ = 1:167. Before filling the model, the total volume and total mass of each layer was calculated according to the strength ratio number, as shown in Table 3.

### 3.3. Set-Up of the Similarity Model

**Figure 3:**
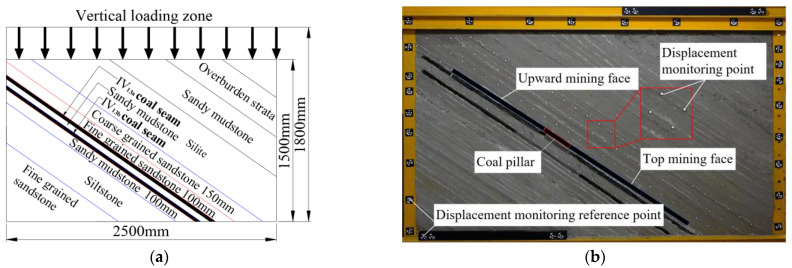
Construction of similar simulation experiment model. (**a**) Model design and size; (**b**) laying model.

The characteristics of a stratum structure and the dip angle of coal mining faces 12031 and 12032 were selected to be references. The following procedure was applied to set up the similarity model: (1) the inner frame of the model frame was rotated by 36°; (2) the loading process was carried out according to the stratigraphic sequence. In accordance with the actual thickness of coal seams in VI_13a_ and VI_13b_, the coal seam thickness of the model was 2.5 m and 3.0 m, respectively. The thickness of sandy mudstone between coal seams was 0.85~4.2 m. In order to bury the pressure sensor, the thickness of sandy mudstone in the experimental model was 5.0 m. A similar model was created using the coal seam floor and coal seam roof, with a total of 12 layers, including a 101.0 cm thick floor rock, 3.0 cm thick VI_13b_ coal seam, 5.0 cm thick sandy mudstone, 2.5 cm thick VI_13__a_ coal seam, 10 cm thick coal pillar, and 163.0 cm thick overburden. The design and laying of the experimental model are shown in Figure 3.

According to existing practical experience, when the model is filled, the thickness of a single layer exceeds 2 cm, which means it is easy for the same layer to become dense and loose and the material to be unevenly laid. At the same time, if the thickness of the single layer is less than 0.5 cm, it is more difficult to form the model. For this reason, in the process of model building, the filling thickness of a single layer is generally 0.5 cm to 2.0 cm, and mica powder is spread as the bedding boundary. In order to prevent gypsum solidification before the model was filled and prevent the strength of similar materials from being affected, similar materials in each layer were mixed uniformly according to the mass ratio, and the filling and compaction were completed within 8 min.

According to the buried depth of the test working face of about 460 m to 585 m, the loading load of the overlying strata was estimated according to the stratum thickness of 500 m, and the loading load was 12.25 MPa. According to the stress similarity constant of 1:167, a load of 73.35 KPa was applied on top of the model using the servo loading system.

**Figure 4:**
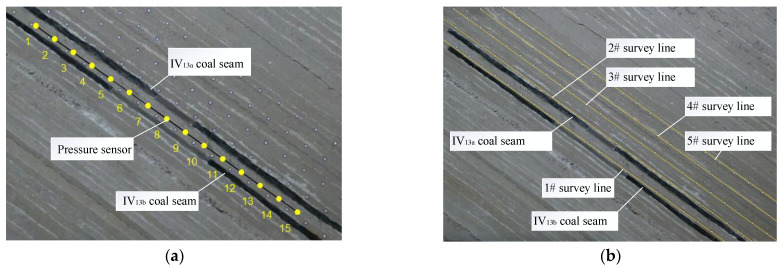
Layout of pressure sensor and measuring line. (**a**) pressure sensor layout; (**b**) schematic diagram of survey line.

In order to monitor the change in floor stress in the mining process of coal seam, 15 pressure sensors were buried in sandy mudstone 2.5 cm below the VI_13a_ coal seam. The measuring points of the 15 pressure sensors were numbered from 1 to 15, with an interval of 10 cm and a distance of 50 cm between the pressure sensors and the boundary on both sides, as shown in Figure 4a.

To determine the distribution characteristics and the migration law of the overburden strata, five survey lines were assigned on the surface of the overlying strata of the model, numbered as 1#~5# survey lines, and there were 123 survey points in total. Survey line 1# was located between the two layers of coal to observe the displacement of the basic roof and the overlying strata during the mining of the lower coal. When the working face was advanced and stabilized, it was photographed and recorded, and the overburden displacement was measured with a three-dimensional photogrammetry system, as shown in Figure 4b.

## 4. Fracture Characteristics of Mining-Induced Overburden

**Figure 5:**
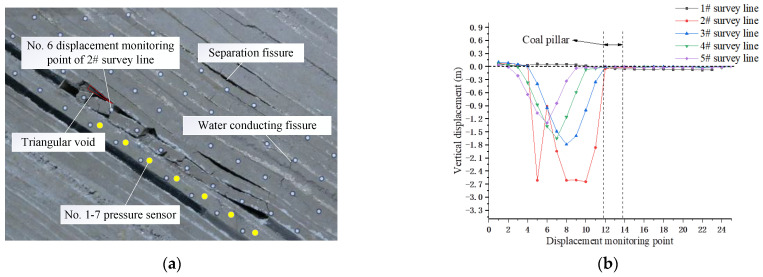
Migration characteristics of overburden in overhead mining. (**a**) Mining 70 m in the upward mining face; (**b**) vertical displacement of overburden in overhead mining.

Both sides of the coal pillar of the upper coal seam (VI_13a_) were excavated using upward mining and downward mining, respectively, as shown in Figure 3b. The 10.0 cm coal seam was excavated at the left side of the coal pillar in the reserved section as the cut hole. The model excavation was then carried out according to the time similarity constant C_t_ (1:10). The 4.8 cm thickness coal seam was mined by inclining upward to facilitate the full settlement of the overburden within 144 min [38], and the mining height was 3 cm. With the advance of the upper coal face, cracks started to appear in the overlying strata, and the direct roof gradually broke and collapsed. After the working face advanced for a certain distance, the basic roof bent and sunk, and separation cracks and water diversion cracks began to appear. The height of separation cracks further developed upward, and the underlying overburden was compacted again to form “three longitudinal zones”. When the working face was excavated to 70 m, it broke and collapsed directly, and the upper rock stratum produced separation fractures. The basic roof collapse slid and fell, and the lower part of the goaf was filled, the middle part was filled, and the upper part was suspended, and a triangular goaf was formed in the upper part of the goaf, as shown in Figure 5a.

The overall trend of the downward movement of the overburden is that the lower displacement was greater than the upper displacement, while the maximum displacement point was affected by the large dip angle and moved along the dip. The displacement of the 2# displacement monitoring point No. 6 of the survey line was significantly reduced compared with the vertical displacement of the measuring points on both sides. It is believed that with the advance of mining in the working face, the direct roof broke and lost stability, and measuring point No. 6 rotated with the sliding block of the basic roof, as shown in Figure 5b.

**Figure 6:**
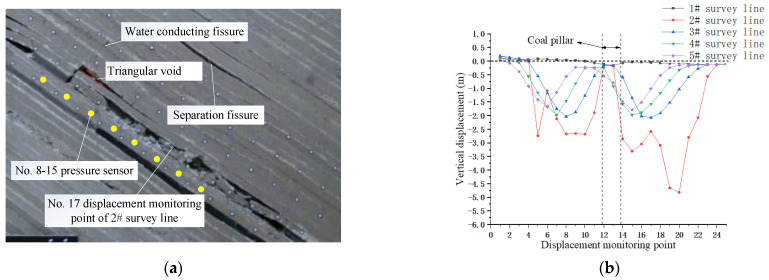
Migration characteristics of overburden under mining. (**a**) Mining 90 m in the top mining face; (**b**) vertical displacement of upper coal overburden.

When the working face advanced to 90 m, obvious separation cracks could be seen from the overburden, and water diversion fracture zones were formed on both sides during this process. The height of separation cracks further developed upward, and the underlying overburden was compacted again, as shown in Figure 6a. Since the first survey line and the survey point above the coal pillar were located at the upper coal floor, there was no obvious displacement. It can be seen from other survey lines that the displacement of the lower side line was greater than that of the upper survey line. That is, the vertical displacement of the lower overburden was greater than that of the upper overburden. The displacement of displacement monitoring point No. 17 of 2# survey line was significantly reduced compared with the measuring points on both sides, as shown in Figure 6b. Cause analysis: direct jacking was broken and unstable, and displacement monitoring point No. 17 slid and rotated with basic jacking.

**Figure 7:**
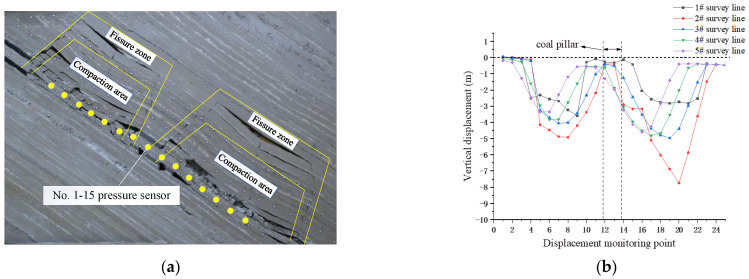
Migration characteristics of overburden under repeated mining. (**a**) Distribution of overburden under double pressure relief; (**b**) vertical displacement of overburden under double pressure relief.

When the overburden collapsed asymmetrically, the caving gangue slipped and was filled unevenly, associated with the collapse of the composite roof. The first breaking steps of the basic roof in inclined mining and inclined mining were basically the same, which were 32.4 m and 32.8 m, respectively. The average periodic breaking step distance of upward mining and downward mining were 13.8 m and 11.7 m, respectively. The periodic breaking step distance of upward mining was significantly greater than that of downward mining. Due to the existence of extremely thick and hard strata, the upper part formed a separation layer suspension space after mining. With the advance of mining in the working face, the separation fractures and their height gradually developed upward, and the lower separation fractures were gradually compacted. The fractures in the upper fracture area were mainly interlayer fractures. The surrounding fracture area formed a fracture area connecting the cutting hole and the working face. It extended in the direction of collapse angle, forming water diversion fractures on both sides of the working face. The overburden fractures were perpendicular to the working face and were distributed in “M”, as shown in Figure 7a.

The deeper coal was excavated using downward mining, and the 10.0 cm coal seam was excavated on the right side of the coal pillar in the reserved section as a cutting hole. Every 144 min, downward mining moved 4.8 cm along the inclined direction of the coal seam, and the mining thickness was 3 cm. With the excavation of the lower coal seam, the vertical displacement curve of overburden had a “W” shape. The subsidence of the top mining face on the 3# survey line (main roof) reached the maximum of 4.06 M about 46.2 m away from the coal pillar. The subsidence of the top mining face on the 3# survey line (main roof) reached the maximum of 4.97 M about 44.5 M away from the coal pillar. The vertical displacement of overburden was significantly greater than that of the top mining face, and the maximum vertical displacement of the top mining face and the top mining face deviated to the depth along the dip angle, as shown in Figure 7b.

## 5. Distribution Law of Mining-Induced Floor Stress

### 5.1. Distribution Law of Floor Stress in Inverted Mining Face

**Figure 8:**
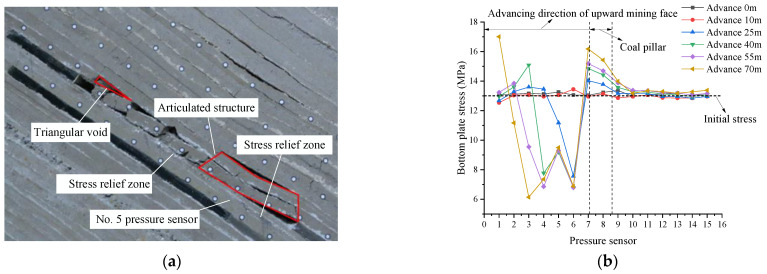
Stress distribution of bottom plate of overhead mining. (**a**) Mining 70 m in the upward mining face; (**b**) stress diagram of top mining floor.

In this section, the stress distribution law of the floor during the excavation of the upper coal seam (VI_13a_) is analyzed. When the 10.0 cm thickness coal seam was excavated on the left side of the coal pillar in the reserved section as the cut hole, the model excavation was carried out according to the time similarity constant C_t_ (1:10). As a result, the 4.8 cm coal seam was mined upward within 144 min, with the constant mining height of 3 cm. The rotation of the basic roof fault rock block formed a stable articulated structure. The goaf was not completely filled, and a triangular goaf was formed in the upper part of the goaf attributed to the large dip of the coal seam. The overburden distribution of the upward mining face is shown in Figure 8a.

It can be seen from Figure 8b that the initial floor stress of shallow buried part (pressure sensor No. 1) was 12.96 MPa, and that of deep buried part (pressure sensor No. 15) was 14.93 MPa. The magnitude of the initial stress was controlled by the loading load and the rock formation at different heights above the pressure sensor. With the advancement in mining of the working face, the bottom plate of the goaf was depressurized in turn, and the stress concentration occurred at pressure sensors No. 7 and 8 (below the coal pillar). The bottom plate stress reached the maximum at pressure sensor No. 7, with a peak value of 17.02 MPa. When the top mining face advanced to 70 m, the bottom plate of the top mining face formed a stress concentration at pressure sensor No. 5, which was quite different from that on both sides, and a stress release area was formed on both sides.

As the advancing length of the working face increased and the initial caving step was reached, the direct roof broke and caved and rotary instability occurred, and a supporting structure was formed about 24 m away from the coal pillar, forming a relatively stable articulated structure. There was an obvious difference compared with the two sides (i.e., the left and right side 10 m and 30 m apart from the coal pillar), resulting in the stress concentration of the bottom plate and the formation of stress release areas on both sides, as shown in Figure 8b.

### 5.2. Distribution Rule of Floor Stress in Underhand Working Face

**Figure 9:**
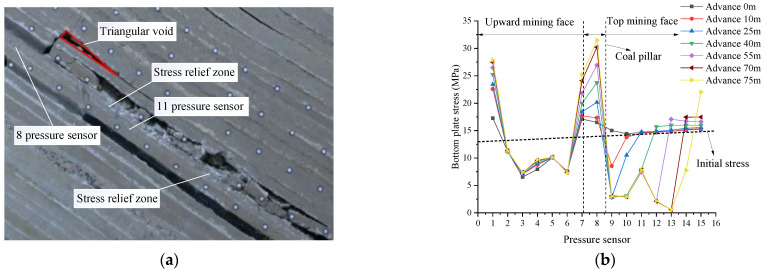
Stress distribution of top mining floor. (**a**) Mining 75 m in the top mining face; (**b**) stress diagram of top mining floor.

The 10.0 cm coal seam was excavated on the right side of the coal pillar in the reserved section as a cut hole. The 4.8 cm coal seam was mined obliquely along the inclined direction of the coal seam within 144 min. After the basic roof was broken for the first time, the basic roof generated rotary deformation on the working face side and the cutting eye side, forming a relatively stable articulated structure, as shown in Figure 9a.

With the advance of mining in the downward mining face, the stress concentration of the bottom plate was further strengthened at pressure sensors No. 7 and No. 8, and the stress concentration appeared at pressure sensor No. 11. The peak stress appeared at pressure sensor No. 8, and the maximum value was 31.50 MPa. The stress of the bottom plate at the side near the overhead mining face below the coal pillar was higher than that at the side of the overhead mining face, and the stress relief degree of the bottom plate of the overhead mining face was higher than that of the overhead mining face, as shown in Figure 9b.

As the advancing length of the working face increased and the initial caving step was reached, rotation instability occurred due to the breakage of the direct roof. When the downward mining working face advanced to 75 m, a support structure was formed at about 27 m from the working face to the coal pillar, resulting in the floor stress concentration. The stress concentration under the coal pillar reached the maximum. Due to the influence of the formation dip angle, the stress release areas on both sides were asymmetric. The maximum stress release value of the overhead mining face (14.07 MPa) was about 2.40 times that of the overhead mining face (5.86 MPa). Considering the fragmentation degree of the surrounding rock, the stress release area 5–19 m away from the coal pillar was found to be more suitable for arranging the lower coal mining roadway.

### 5.3. Distribution Characteristics of Floor Stress

**Figure 10:**
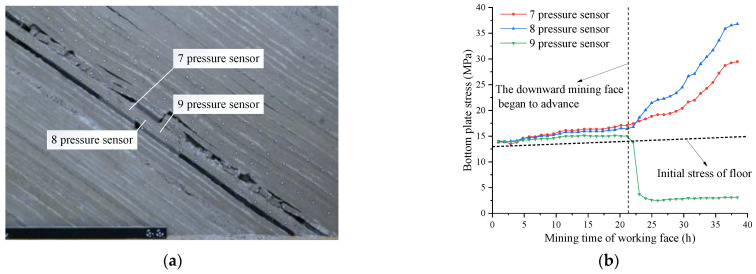
Variation of floor stress during upper coal mining. (**a**) Overlying rock displacement of upper coal mining; (**b**) stress variation diagram of bottom plate.

After the upper coal was recovered, some radial fractures in the upper layer of the basic roof were closed, mainly attributed to the sliding and filling effect of caving gangue. Note that there was no obvious change in the height of the caving zone. During the continuous advancement of the working face, the separation fractures and separation heights of the basic roof overburden gradually developed upward, forming a curved subsidence zone. Even so, there were no obvious through radial fractures, as shown in Figure 10a.

The overburden pressure was transmitted downward through the coal pillar and the coal walls on both sides with the excavation of the upper coal seam. In order to understand the stress distribution state under the coal pillar, pressure sensors No. 7, 8 and 9 located under the coal pillar need further analysis. With the advance of mining in the working face, the floor stress at pressure sensors No. 7 and No. 8 gradually increased. After the upper coal mining, the floor stress at No. 7 was 29.47 MPa, and that at No. 8 was 36.80 MPa. The floor stress at pressure sensor No. 8 was significantly higher than that at No. 7, which was generally affected by the large dip angle. With the increase in the mining advance distance of the working face, the bottom plate stress of other measuring points was relieved in turn. In detail, the bottom plate stress release at pressure sensor No. 9 reached the maximum, and the minimum value was reduced to 2.49 Mpa, which was 17.7% of the initial stress, as shown in Figure 10b.

With the advance of the working face, the basic roof of the upper coal seam with a large dip angle broke and collapsed, and the overlying strata bent and sunk. Due to the dip angle of the coal seam, the vertical displacement of the overburden in the deep working face was greater than that in the shallow working face. The stress was transmitted downward along the coal pillar and coal wall. The asymmetric migration of overburden led to the asymmetric distribution of stress in the coal pillar and the floor on both sides. The side stress of the coal pillar floor near the deep working face was more concentrated, and the stress under the goaf of the deep working face decreased more significantly than that of the shallow working face.

**Figure 11:**
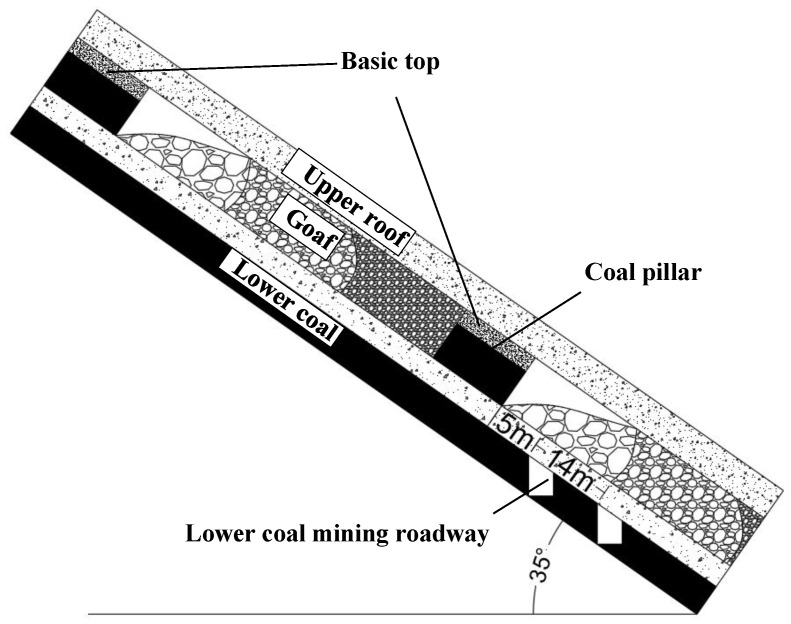
Layout of roadway under residual coal pillar in large-dip seam.

Arranging a mining roadway in the low-stress area below the goaf could effectively reduce the construction time and support costs. It is thus highly recommended to arrange a lower coal mining roadway below the goaf of the deep working face in the low-stress area 5–19 m away from the coal pillar. The layout of the roadway under the residual coal pillar in the large-dip coal seam is shown in Figure 11.

## 6. Results

A systematic similarity simulation was conducted based on the production and geological conditions of Zhongwei coal mine with a typical large-dip coal seam. The stress distribution characteristics of the upper coal floor and the law of overburden displacement with upward mining and downward mining were analyzed. The main conclusions of this research are listed as follows:

(1) The collapse and breakage of the basic roof of the upper coal seam with a large dip resulted in a triangular goaf. The overburden fracture area was perpendicular to the working face and presented “**M**“ distribution with the excavation of the lower coal seam, whereas the vertical displacement of the lower coal overburden presented “W” distribution with double pressure relief;
(2) The vertical displacement of overburden in the top mining face was generally greater than that in the upward mining face. With the advance of the working face, the maximum displacement shifted to the deep seam along the dip angle;
(3) With the increase in the mining advance distance, floor stress concentration occurred about 24 m (upward mining) and 27 m (top mining) from the coal pillar in the upward mining and top mining working faces, respectively. The distance from the top mining stress reduction area to the coal pillar was generally greater than that in the upward mining working face;
(4) Affected by the stratum dip angle, the displacement of deep overburden was greater than that of shallow overburden. The floor stress under the coal pillar was asymmetrically distributed. The pressure relief degree of the floor stress of the deep working face was higher than that of the shallow working face. The asymmetric movement of overburden led to the differential distribution of floor stress;
(5) In order to facilitate roadway support and save costs, a lower coal roadway is recommended to be arranged below the goaf 5–19 m away from the coal pillar in the deep working face.

## Figures and Tables

**Table 1:** Lithologic characteristics of roof and floor of VI_13_ coal seam.

Name of Top and Bottom Plate	Rock Category	Thickness (m)	Lithology Description
Overlying strata	Coarse-grained sandstone	21.60	Medium-thick, layered sandy conglomerate, gravelly coarse sandstone and quartz coarse sandstone
Basic top	Fine-grained sandstone	10.10	Layered, fine coarse sandstone
Pseudo top	Carbonaceous mudstone	0.57	Thin-layer carbonaceous mudstone
Direct bottom	Carbonaceous mudstone and fine sandstone	10.95	Mainly carbonaceous mudstone and fine sandstone, locally sandy mudstone, with small cross bedding
Basic base	Siltstone, fine–medium–coarse sandstone	24.90	Siltstone, fine–medium–coarse sandstone, locally carbonaceous argillaceous siltstone

**Table 2:** Main rock mechanical strength of roof and floor of VI_13_ coal seam.

Rock Name	Compressive Strength (MPa)	Tensile Strength (MPa)	45° Shear Strength (MPa)	Elastic Modulus (GPa)
Coarse sandstone	57.82	2.86	30.74	11.30
Fine sandstone	64.15	3.09	34.15	12.74
Carbonaceous mudstone	18.03	2.05	9.33	4.16
Coal	6.35	0.39	1.20	0.52
Sandy mudstone	27.37	2.65	13.27	4.10
Fine sandstone	72.38	3.57	38.62	14.35

**Table 3:** Proportion and dosage of similar materials.

Serial Number	Lithology	Actual Thickness (m)	Model Thickness (cm)	Matching Number	River Sand (Kg)	Calcium Carbonate (Kg)	Plaster (Kg)	Water (Kg)	Remarks
1	Overlying strata	62.83	64.00	673	42.27	4.93	2.11	7.05	
2	Sandy mudstone	25.00	25.00	673	224.61	26.20	18.23	37.44	
3	Siltstone	42.87	43.00	673	457.02	53.32	22.85	76.17	
4	Coarse sandstone	21.60	21.00	646	187.48	12.50	18.75	31.25	
5	Fine sandstone	10.10	10.00	737	120.74	5.17	12.07	19.71	Basic top
6	VI_13a_ coal seam	1.70~2.70	2.50	837	34.74	1.3	3.04	5.58	
7	Sandy mudstone	0.85~4.20	5.00	673	53.79	6.28	2.69	8.79	
8	VI_13b_ coal seam	2.50~3.20	3.00	837	32.20	1.21	2.82	5.18	
9	Carbonaceous mudstone	4.10	4.00	673	96.90	11.30	4.84	16.15	Direct bottom
10	Fine sandstone	6.85	7.00	737	84.52	3.62	8.45	13.80	
11	Basic base	90.20	90.00	346	362.26	48.30	72.45	48.30	

## Data Availability

The research data used to support the findings of this study are currently under embargo while the research findings are commercialized. Requests for data, 12 months after publication of this article, will be considered by the corresponding author.
